# Absence of reproduction-immunity trade-off in male *Drosophila melanogaster* evolving under differential sexual selection

**DOI:** 10.1186/s12862-019-1574-1

**Published:** 2020-01-28

**Authors:** Zeeshan Ali Syed, Vanika Gupta, Manas Geeta Arun, Aatashi Dhiman, Bodhisatta Nandy, Nagaraj Guru Prasad

**Affiliations:** 1 Department of Biology, Syracuse University, 110 Life Sciences Complex, 107 College Place Syracuse, Syracuse, NY 13244 USA; 2 Department of entomology, Cornell University, 3130 Comstock Hall, Ithaca, NY 14853 USA; 3 Department of Biology, Indian Institute of Science Education and Research Mohali, Sector 81, SAS Nagar, Mohali, 140306 India; 4 Kalahari Molerat Project, Kuruman river reserve, Northern cape, SA South Africa; 5 Department of Biology, Indian Institute of Science Education and Research Berhampur, Govt. ITI Building, Engg. School Junction, Berhampur, 760010 India

**Keywords:** Sexual selection, Immunity, *Drosophila melanogaster*, Trade-off

## Abstract

**Background:**

The theory of trade-off suggests that limited resources should lead to trade-off in resource intensive traits such as, immunity related and sexually selected traits in males. Alternatively, sexual exaggerations can also act as an honest indicator of underlying immunocompetence, leading to positive correlations between these traits. Evidences in support of either hypothesis in invertebrates are equivocal. Whereas several studies have addressed this question, few have used naturally occurring pathogens and realized post infection realized immunity (e.g., survivorship) to assay the fitness correlations between these two sets of traits.

**Results:**

Adopting an experimental evolution approach, we evolved replicate populations of *Drosophila melanogaster* under high and low sexual selection regimes for over a hundred generations and found the following in virgin and mated males in three separate assays:

- Post infection survivorship against two natural pathogens - *Pseudomonas entomophila* (Pe) and *Staphylococcus succinus* (Ss): Mated males survived better against Pe, but were no different than virgins against Ss.
- Bacterial clearance ability against a third natural pathogen *Providencia rettgeri* (Pr): Mated males had significantly lower CFUs than virgins.

However, sexual selection history had no effect on realized immunity of either virgin or mated males.

**Conclusion:**

We show that while mating can affect realized immunity in a pathogen specific way, sexual selection did not affect the same. The results highlight that complex polygenic traits such as immunity and reproductive traits not necessarily evolve following a binary trade-off model. We also stress the importance natural pathogens while studying sexual selection-immunity correlations.

## Background

Two of the most important sets of traits that determine a male’s fitness are sexually selected traits and immunity related traits. Both are resource intensive in their maintenance and deployment and, as life history theory suggests, are expected to trade-off with other life history related traits as a consequence [1]. Traits such as longevity, stress resistance and fecundity have been shown to trade-off with both immunity [2–5] and sexually selected traits [6]. Such trade-offs are widespread, although not universal [7–9], and are important in our understanding of the maintenance of variation in life history traits in the face of strong directional selection.

Following the above argument, sexually selected and immunity related traits are also expected to trade-off with each other. Additionally, in males, such trade-offs can be apparent only with reproductive effort, because several traits under sexual selection (such as courtship display and mating calls) manifest in the specific context of mating. Populations evolving under differential levels of sexual selection can evolve differential levels of reproductive investment during mating [6, 10–13]. This difference might result in differential effect of mating in their response to pathogenic infections. Alternatively, Hamilton and Zuk proposed that male sexual traits might reflect their underlying immunocompetence, and therefore the two sets of traits are likely to be positively correlated [14]. Studies addressing genetic correlation between mating and immunity in vertebrates have been the focus of much research following this pioneering work [15, 16].

Due to the relatively simple immune system and small generation time of many invertebrate model organisms, it is possible to design tractable experimental evolutionary studies to test the alternative hypotheses [17]. Phenotypic correlation between reproductive investment in males and several components of immunity has been studied in many invertebrate species. In wolf spiders, males presented with females increase their drumming rates at a cost of lytic activity (LA) [18]. Negative correlations between encapsulation rate (EN) and both call syllable number and spermatophore size were shown in bush crickets [19]. In decorated crickets, artificial induction of spermatophore production traded off with phenol oxidase activity (PO) and LA [20], and induction of immune system through lipopolysaccharide injection resulted in the reduction of their daily call rate [21]. In a more direct assay of immunological cost of mating, McKean and Nunney showed that increased sexual activity decreased the ability to clear the non-pathogenic bacteria *E. coli* by male *Drosophila melanogaster* [22]. Conversely, Gupta et al. found that mating increased the ability to survive infection and clear the natural pathogen *Pseudomonas entomophila* in males from three unrelated populations of *D. melanogaster* [23]. Similar results have also been found in bumblebees [24] and mealworm beetles [25].

The evolutionary relationship between sexually selected traits and immunity, at least in invertebrates, is equivocal. Simmons et al. (2010) calculated quantitative genetic variation in immunity related and sexually selected traits in the Australian cricket *Teleogryllus oceanicus* using half-sib analysis and found a negative genetic correlation between these two sets of traits [26]. Mckean and Nunney, using experimental evolution, altered the intensity of sexual selection in laboratory populations of *Drosophila melanogaster* by skewing the sex ratio towards males [27]. Higher sexual selection imposed on males resulted in lesser ability to clear the bacteria *E. coli*. In the yellow dung fly, *Scathophaga stercoraria,* removal of sexual selection through monogamy resulted in increased PO activity but that did not translate into increased antibacterial effect in vitro [28]. In the flour beetle, *Tribolium castaneum,* similar removal of sexual selection did not result in difference in either PO activity or their ability to survive the infection by the pathogenic microsporidian *Paranosema whitei* [29].

A recurring theme in many of the above mentioned studies, as observed by Lawniczak et al., is the lack of a fitness oriented experimental framework [17]. Changes in molecular parameters of immune response (such as gene expression, PO and LA) do not always translate into fitness differences (e.g., [29]). This leads to a dissonance between potential (gene expression, PO, LA etc.) and realized (actual ability to survive pathogenic infection)immunity [30].Experimental evolution is a promising framework of addressing the issue wherein evolving host populations under different levels of sexual selection, followed by fitness measurements (e.g., survivorship) against pathogenic infection can help us directly assess the correlation between sexual selection and realized immunity. That said, even the supposedly simple immune system of invertebrates is in fact not that simple, with several studies showing pathogen specificity [31], immune memory [32], and transgenerational immune priming [33]. The pathogen(s) that a host is exposed to constitute an important part of the host’s ecological context and can play a non-trivial role in determining the outcome of the interaction between reproductive investment and realized immunity. If the same host responds through different immune mechanisms to different pathogens (i.e., specificity), mating may have differential effect on host ability to combat different infections. For example, Gupta et al. showed that males from the same populations of *D. melanogaster* which showed increased resistance against *P. entomophila* upon mating did not show any effect of mating when challenged with *Staphylococcus succinus* [23]. This argument can be extended to the evolutionary effect of sexual selection in males on their immune response as well. Therefore, in order to assess these relationships, it is important to measure host fitness against different ecologically relevant pathogens. However, such studies are rare.

In this study, we try to address this issue by evolving replicate populations of *Drosophila melanogaster* under increased and decreased levels of sexual selection for more than a hundred generations. Alteration of sexual selection was achieved by maintaining the populations under female biased (F) or male biased (M) operational sex ratio regimes. Previous studies have shown that males in these populations have diverged in terms of their reproductive traits, such as courtship and locomotor activity, and sperm competitive ability [6, 10]. We subjected the males from both regimes to infection by three ecologically relevant bacteria –*Pseudomonas entomophila* (Pe), *Staphylococcus succinus* (Ss), and *Providencia rettgeri* (Pr) in three different assays. To address the effect of mating, in each of the assays, we had two groups of males from each selection regime – virgin and sexually active. We used survivorship post infection as a measure of fitness in two of the assays (Pe and Ss), and ability to clear bacteria in the third (Pr). For the assay with Pr, we further quantified the number of mating and courtship for sexually active M and F males. Using this framework, we tested whether:

- There is an effect of sexual selection (M vs F), mating activity (virgin vs sexually active) or their interaction upon realized male immunity when challenged by a natural pathogen.
- Such effects are pathogen dependent or consistent across different pathogen.
- Variation in mating and/or courtship activity is reflective of variation in pathogen clearance ability.

## Results

We collected virgin males from F and M regimes, each containing three independent blocks. Two to three-day-old males were divided into two groups- virgin (males kept in single sex vials for two days) and mated (males combined with ancestral females for two days). Flies were infected following protocols used in [23] (please see methods for further details).

**Fig. 1:**
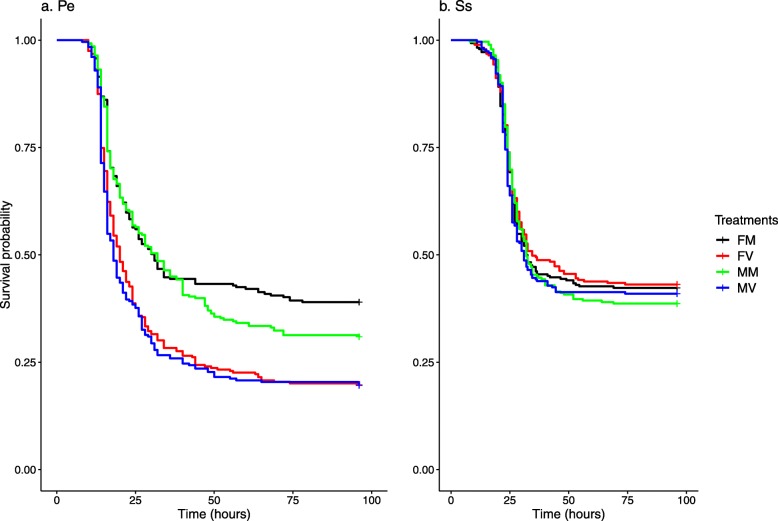
Results of Cox proportional hazards analysis for survivorship against: (**a**)*Pseudomonas entomophila* and (**b**) *Staphylococcus succinus*. The curves show survival as a function of time. The black, green, red and blue lines represent F-mated (FM), M-mated (MM), F-virgin (FV) and M-virgin (MV) respectively

For survival analysis, we compared the Cox partial likelihood (log-likelihood) estimates. Mating had a significant effect on survival against Pe (Table1a). Pairwise comparisons showed that mated males survived better than virgins in both F and M regimes (*p* < 0.001, Fig. 1a). However, there was no effect of selection or selection × mating status interaction. There was no effect of either mating, selection regime or selection × mating interaction on survivorship against infection by Ss (Fig. 1b, Table 1b).

**Table 1:** Analysis of cox proportional hazards for survivorship post-infection for (A) *Pseudomonas entomophila* and (B) *Staphylococcus* succinius, and analysis of bacterial colony count data (natural log transformed) against *Providencia rettgeri* (C), Significant effects are marked in bold

A. Survivorship against *Pseudomonas entomophila*						
	loglik		Chisq	Df	Pr(>|Chi|)	
Selection	− 5035.2		0.9701	1	0.325	
Mating_status	− 5011.8		46.8518	1	**7.656e-12**	
Selection × Mating_status	−5011.6		0.2909	1	0.59	
B. Survivorship against *Staphylococcus succinus*						
Selection	− 4377.6		3.4314	1	0.361	
Mating_status	−4377.6		0.0556	1	0.870	
Selection × Mating_status	−4377.5		0.1624	1	0.675	
C. Bacterial clearance ability against *Providentiarettgeri*						
	Sum Sq	Mean Sq	NumDF	DenDF	F.value	Pr(>F)
Selection	0.447	0.447	1	4.7040	0.0986	0.766948
Mating_status	60.619	60.619	1	15.3587	13.3857	**0.002249**
Selection× Mating_status	0.126	0.126	1	7.0301	0.0279	0.872035

**Fig. 2:**
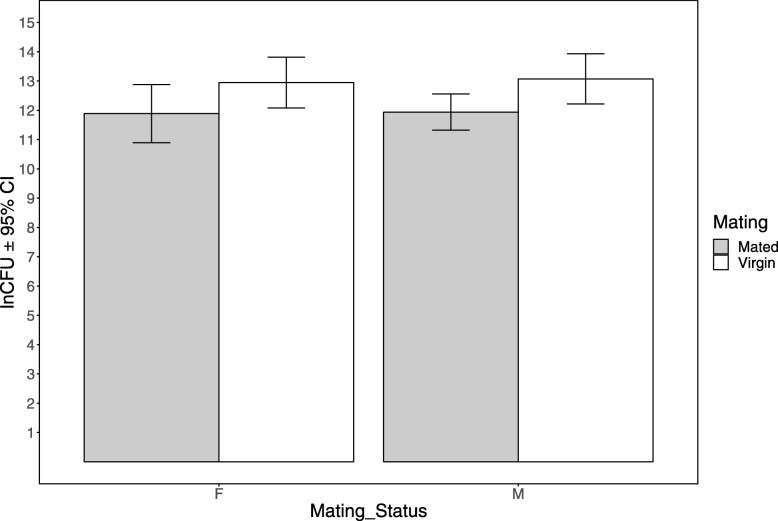
Results of natural log transformed CFU data for mated (Shaded bar) and virgin (open bar) treatments of M and F regimes which are represented in the x-axis. The error bars represent 95% confidence intervals. In both selection regimes mated males had significantly lower colony count than virgins

**Fig. 3:**
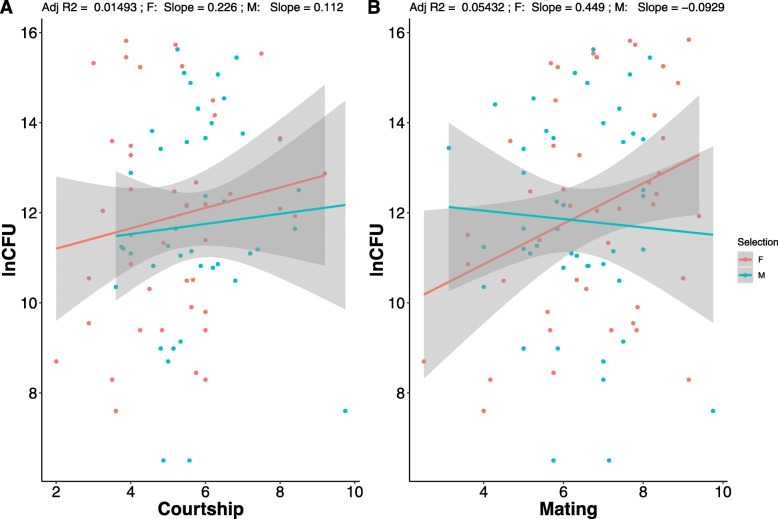
Correlation plots of bacterial load (log transformed CFU numbers) and (**a**) number of mating and (**b**) amount of courtship. Green and red points represent vial averages for the pair of traits in M and F regime respectively

In the assay where Pr was used as a pathogen, flies were homogenized in MgSO4 and plated using a robotic plater. After incubating overnight CFUs were measured. There was no difference between F and M males in their average number of mating (*p* = 0.7872, Additional file 1: Fig. S1), whereas M males directed more courtship towards females than F males (*p* = 0.013, Additional file 2: Fig. S2). For the CFU data, we found a significant effect of mating, but no selection × mating interaction effect (Table 1c). Post-hoc analysis showed that mated males were able to clear more bacteria compared to virgins in both F and M regimes (Fig. 2). Regression models showed that variation in neither the number of mating nor the amount of courtship explained the variation in CFUs (Fig. 3a, b).

## Discussion

The evolutionary and phenotypic relationship between male immunity and reproduction, especially in invertebrates, has been a debatable issue with equivocal results [17, 34]. We attempt to contribute to this body of studies using experimentally evolved replicate populations of *Drosophila melanogaster* and measuring their post-infection realized immunity against three different natural pathogens – *Pseudomonas entomophila* (Pe), *Providentia rettgeri* (Pr) and *Staphylococcus succinus* (Ss).

Taken together, the results show that:

- In this system, sexual selection did not affect realized immunity post infection against any of the three pathogens used in the present study.
- The act of mating had a positive effect on realized immunity in a pathogen specific manner. However, the number of mating or amount courtship did not explain this positive effect.

### No effect of sexual selection on immune response

Within mating treatment, males from M and F regimes did not differ from each other in terms of either post infection survivorship (against Pe and Ss) or bacterial clearance ability (against Pr). Our results differ from those of a previous study which measured host’s ability to clear *E.coli* as a proxy for immune response, and found a trade-off with the intensity of sexual selection [27]. This difference shows that the relationships between multi-locus traits such as immunity related traits and traits under sexual selection can be complex and may not follow a simplified binary model of trade-off [1]. Several other studies have measured one or a few component(s) of immunity, such as phenoloxidase activity and found them to be negatively correlated with the intensity sexual selection [28, 29]. However, studies that measure one (or a few) component(s) of immunity to assay the effect of sexual selection on immunity can have certain drawbacks. Different components of the immune system can have their own internal correlations. For example, a negative genetic correlation between resistance and tolerance has been reported in a mouse-*Plasmodium chabaudi* system [35]. Within-immune system trade-offs have also been found in female white-footed Mice, *Peromyscus leucopus* [36]. Therefore, measuring just one or a few components can lead to incomplete and perhaps misleading conclusions about the genetic correlations between immunity and sexual selection. Furthermore, some of these components might have no fitness consequence. A study found increased PO activity in males did not alter their antimicrobial activity in yellow dung fly (Hosken, 2001). Further, Leclerc et al. found that in *Drosophila melanogaster*, mutants that failed in producing active phenoloxidase had equal survivorship compared to wild type flies against pathogenic infection by different species of fungi and, both gram positive and negative bacteria indicating redundant immune pathways for survival against a wide variety of microbes [37]. Thus, while measuring components of immunity is important to understand the functional basis, its fitness consequence would ultimately drive the evolution of the trait, and it is therefore important to measure immunity in that context. In the present case, we have used three different natural isolates of bacterial pathogens of *D. melanogaster* and showed that neither survival nor bacterial clearance ability changes in response to differential levels of sexual selection, suggesting that in this system, response to sexual selection has not been traded-off with investment in overall immune response. A putative explanation is that the average number of mating acquired by the males from the two different selection regimes is not significantly different. Thus if the effect of selection depends upon mating activity (such as that in [27]), and not upon investment per mating, the effect of reproduction upon immunity is expected to be the same between the two selection regimes. The effect of reproduction on immunity in our experiment is discussed in the next section.

### Phenotypic effect of reproduction on immunity depends on pathogen

We found that mated males from both M and F regimes had better survivorship and bacterial clearance abilities against Pe and Pr. We have previously shown that in the population ancestral to the selection lines used here, mating had a beneficial effect on resistance against Pe [23]. Our results also corroborate other studies which found that mating can be beneficial against infections [24, 25]. However, these results differ from those of McKean and Nunney, who found trade-off between mating and immunity in terms of bacterial clearance [22].

Additionally, it has been proposed that courtship effort by males can affect post-mating immune response. However, in this study variation in both mating and courtship effort failed to explain the variation in bacterial clearance ability of males against Pr, as evidenced by the lack of correlation between average courtship and CFU (Fig. 3a). This was true for both M and F regime males. Furthermore, while M males courted females more than F males (both in the ‘Mated’ treatment), their bacterial clearance ability were not different. Thus, it seems likely that the qualitative change in mating status is more important for the observed change in realized immunity than the quantitative variation in either number of matings or amount of courtship in this system.

Phenotypic relationships between multi-component traits such as immune response (with mutually non-exclusive components such as resistance, tolerance, memory etc.) and reproduction (with components such as acquisition of mates, production of sperm and accessory gland proteins etc.) are expected to be complex – even invertebrates like fruit flies show great variety and pathogen specificity in their response to infections. Thus, measuring such relationships is expected to depend upon the pathogens. The fact that we find no difference in survivorship between mated and virgin males against Ss further highlights the issue.

### Evolutionary response does not mirror phenotypic correlation

McKean and Nunney showed that increased sexual selection resulted in evolved populations of *Drosophila melanogaster* where males had exaggerated sexually selected traits, but had reduced ability to clear the non-pathogenic bacteria *E. coli*. This result mirrored the phenotypic trade-off they found between mating and immunity [22, 27]. Our results differ from that of McKean and Nunney in that we found mated males to have higher survivorship and bacterial clearance ability against Pe and Pr respectively whereas males from both M and F regimes had equal ability to survive infection or clear bacteria within a given mating treatment. Thus, our results did not show a mirroring of the genetic and evolutionary relations found by McKean and Nunney. The most likely explanation is that, it is not really necessary for genetic correlations to mirror phenotypic effects [38]. Genetic and phenotypic correlations depend upon various factors such as age, developmental conditions, resource availability etc. [39]. Therefore, these factors might impact the correlation between traits through genotype × environment interactions. For example, genetic correlations between immunity and other life history related traits have been found to be dependent upon the host condition [40] and temperature [41]. Therefore, it is possible that the phenotypic and genetic relations between sexual selection and immune response might manifest in conditions that differ from their maintenance regime.

## Conclusions

Using three different pathogens of *Drosophila melanogaster*, we found no evolutionary effect of the intensity of sexual selection on the immunocompetence of males. This is in contrast with several previous studies [27, 42, 43]. We also show that mating can have beneficial or no effect on males depending upon pathogen. This adds to a growing body of studies that have used natural pathogens to show the beneficial effects of mating on hosts [23–25]. Taken together, our study provides further evidence that the complex life history relationships, such as that between reproductive investment and immune response might not manifest in the form of binary trade-offs, either genetic or phenotypic [44].

## Materials and methods

### Ancestral populations

The two ancestral populations used in this study are called LH and LH_st_, both large laboratory adapted populations of *Drosophila melanogaster*. The LH population was established by Lawrence Harshman from 400 gravid wild caught females. This population is maintained at an effective population size > 5000 [45]. LH_st_ was derived by introgression of a benign autosomal ‘scarlet eye’ marker to the LH genetic background and is maintained at an N_e_ > 2500. The LH and LH_st_ populations are genetically equivalent except for one locus which has no effect on their fitness. The additive genetic variation in the LH_st_ population is maintained through periodic back cross with LH [46]. Both populations are maintained at standard laboratory condition (temperature = 25 °C, relative humidity ≈ 60%) in a 12:12 dark: light cycle and are reared on corn-meal molasses food. Detailed population maintenance is described in [47]. Briefly, in a given generation, 2–3-day-old adult flies from rearing vials (95 mm height × 25 mm diameter) are mixed and redistributed into fresh food vials − 16 males and 16 females in each - containing a limiting quantity of dried yeast granules. The flies are kept there for two days after which they are allowed to oviposit for 18 h in fresh vials with food. These vials are controlled for density (~ 150 eggs /vial) and incubated to start the next generation.

### Selection regimes

The selection regimes are derived from LH_st_. Initially three populations, C_1–3_, were derived and maintained for 5 generations. The maintenance of the C populations differed from that of LH_st_ in that adult males and females were collected as virgins and held in same-sex vials with 8 individuals/vial and combined in 1:1 sex ratio (16 males and 16 females) once they were 2–3 days old with measured amount of live yeast paste instead of granules. Thereafter the maintenance protocol is the same as that of LH_st._ After 5 generations, two more selection regimes, F_1–3_ and M_1–3_, were derived from each of the C populations where operational sex ratios where biased towards males and females respectively. In these populations, 2–3 day-old virgin adults were combined in their respective sex ratios, i.e., Male: Female ~ 1:3 and 3:1 for F and M respectively. Note that the populations sharing the same subscript share a common ancestry and are handled simultaneously, independent of those having a different subscript. Thus, each subscript constitutes a “statistical block”. Details of maintenance and selection history is described in [10].

### Stndardization

Nongenetic parental effects [48]can lead to misinterpretation of multi-generation selection experiment results. To equalize such effects across selection regimes, all selected populations were passed through one generation of standardization where selection was removed, i.e., they were maintained in ancestral conditions [49]. Adult progeny produced by this generation were used for the experiment.

### Bacterial culture

We used three pathogens for this study: gram negative bacteria *Providencia rettgeri* [50], gram negative bacteria *Pseudomonas entomophila* L48 [51], and gram positive bacteria *Staphylococcus succinus subsp. Succinus,* strain PK-1 (Ss) [52]. All three bacteria are natural isolates obtained from wild caught *Drosophila.* For making the bacterial suspension for infections, bacterial culture was grown at 27 °C (Pe) and 37 °C (Ss and Pr) till OD = 1.0 ± 0.1 from a glycerol stock maintained at -80 °C. Following this, cells were pellet down and suspended in equal volume of 10 mM MgSO_4_ before infection. For Pr, the suspension was concentrated to OD 2.0 ± 0.1 before infection.

### Infection protocol

Flies were put under light CO_2_ anaesthesia and infected by pricking with a needle (*Minutein pin* 0.1 mm*,* Fine Science Tools, CA) dipped in bacterial suspension (bacteria suspended in 10 mM MgSO_4_) in the thorax (Gupta et al.2013). To control for injury, a separate set of flies were pricked with a tungsten needle dipped in sterile 10 mM MgSO_4_ (sham).

### Experimental treatments

For each of the three separate assays (using the three pathogens), the following experimental protocol was used:

Experimental males were collected within 6 h of eclosion from pupae, which ensured their virginity, since in these populations it takes the flies ~ 8 h to attain sexual maturity. These males were kept in vials provided with corn-meal molasses food at a density of 8 males /vial. On 12th day post egg collection (i.e., 2–3-day-old adult) flies from each selection regime were randomly assigned to two groups: ‘virgin’ and ‘mated’.

In the ‘virgin’ treatment, virgin males were transferred to vials containing fresh food as they were. In the ‘mated’ treatment, males from each vial were combined with virgin LH_st_ females (8 / vial). A total of 15 vials were set up per treatment per selection regime per block. Ten (*n* = 80) and 5 (*n* = 40) vials were used for infection and sham (control) respectively. All pricking was done on 14th day post egg collection and were transferred to vials containing fresh food following infection. Males in the ‘mated’ treatment were separated from females while anaesthetized for pricking and were maintained in single sex vials.

### Mating and courtship measurements

We measured the number of mating obtained by selection regime males in the ‘mated’ treatment in the assay where Pr was used. All the vials in the ‘mated’ treatment were observed manually. A total of 22 observations were taken over a period of 48 h, with more frequent observations during the light-dark and dark-light transition (+/− 2 h before and after the transitions). Number of mating pairs and courting males were recorded at each observation timepoint. Average number of mating and courting male per vial were calculated and used as the unit of analyses using the following formula:

$$ \sum \limits_{i=1}^{22}\frac{\mathrm{total}\ \mathrm{mating}\ \left(\mathrm{or}\ \mathrm{courting}\right)\mathrm{males}\ \mathrm{during}\ \mathrm{the}\ {i}^{th}\ \mathrm{obs}\ }{\mathrm{number}\ \mathrm{of}\ \mathrm{males}\ \mathrm{in}\ \mathrm{the}\ \mathrm{vial}\ } $$

We used this as a proxy of the total amount of mating acquired or courtship displayed by a male over the period of 48 h.

### Measure of infection response

For Pe and Ss, response to pathogenic infection was measured in terms of survivorship post infection by observing vials for mortality every three hours post infection for ~ 100 h post infection. For Pr, since mortality was low (< 5%) and did not differ from the sham control, response was measured as the ability of the host to clear bacteria using a previously established method [23]. Briefly, 20 h post infection, 6 flies from each vial were sampled randomly and divided into groups of three. They were then crushed using a mortar inside micro-centrifuge tubes containing 100 μL MgSO_4_ and plated on LB-Agar plates using an automated spiral plater (WASP spiral plater, Don Whitley Scientific, UK). Three replicate plates were plated from each group of three flies. After growing the bacteria in their respective optimum temperatures, CFUs were counted using a plate reader (Acolyte colony counter, Don Whitley Scientific, UK). Average CFUs per fly obtained from each group was used as unit of analysis.

### Statistical analyses

All analyses were performed in R. Survivorship (for Pe and Ss) was analyzed using Cox’s Proportional hazards model. Time to death was recorded for each fly and flies not dead till the last time were treated as censored data. For each of the pathogens, data were modelled either using block as a random factor or excluding Block using R package “Coxme ”[53]using the following two expressions:

Model 1: ~ *Selection* + *Mating* _ *status* + *Selection* : *Mating* _ *status* + (1 │ *Selection* : *Block*).

Model 2: ~ *Selection* + *Mating* _ *status* + *Selection* : *Mating* _ *status*

Since analysis of deviance revealed no effect of block(analysis of deviance test: χ^2^_2_ = 0.72, *p* = 0.69 for Pe; χ^2^_2_ = 0.01, *p* = 0.99 for Ss), data from all three blocks were pooled and the cumulative data were then tested for difference in survivorship. We compared the Cox partial likelihood (log-likelihood) estimates across treatments and selection regimes.

In the case of Pr, colony count data was natural log transformed and normality was verified using a Shapiro – Wilk test. To test for various factors data were then subjected to the following glm models using package “lme4” [54] (all relevant R codes are provided as SI):

Effect of selection regime and mating status (mated vs virgin) on CFU:

$$ lnCFU\sim \kern0.5em Selection+ Mating\_ status+ Selection: Mating\_ status+\left(1| Selection: Block\right) $$

Effect of selection regime on mating and courtship:

$$ Mating\left( or\ courtship\right)\sim \kern0.5em Selection+\left(1| Selection: Block\right) $$

Test for the effect of courtship on CFU in mated males belonging to the two selection regimes:

$$ lnCFU\sim \kern0.5em Selection+ Courtship+\left(1| Selection: Block\right) $$

## Supplementary information

**Additional file 1: Fig. S1.** Difference in the amount of courtship between the two selection regimes.

**Additional file 2: Fig. S2.** Difference in the number of matings acquired by the males between the two selection regimes.

## Data Availability

All data generated or analysed during this study are included in this published article [and its supplementary information files].
