# Supplementary figures and images for: Absence of reproduction-immunity trade-off in male Drosophila melanogaster evolving under differential sexual selection
Source: BMC Evol Biol. 2020 Jan 28;20:13. doi: 10.1186/s12862-019-1574-1 (PMC6988192; doi:10.1186/s12862-019-1574-1)

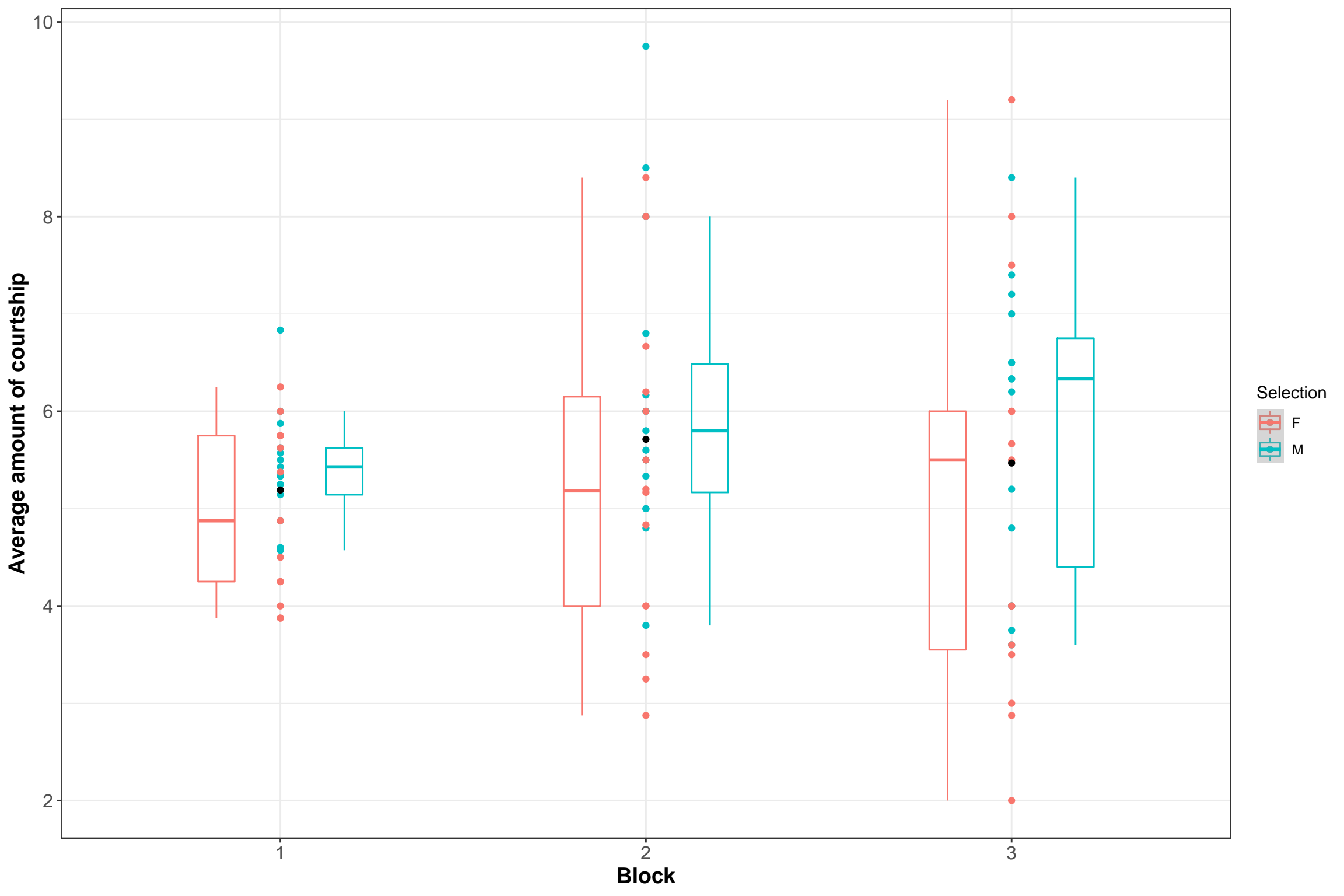

Supplement: Supplementary file 1 — Additional file 1: Fig. S1. Difference in the amount of courtship between the two selection regimes. [file 12862_2019_1574_MOESM1_ESM.pdf]

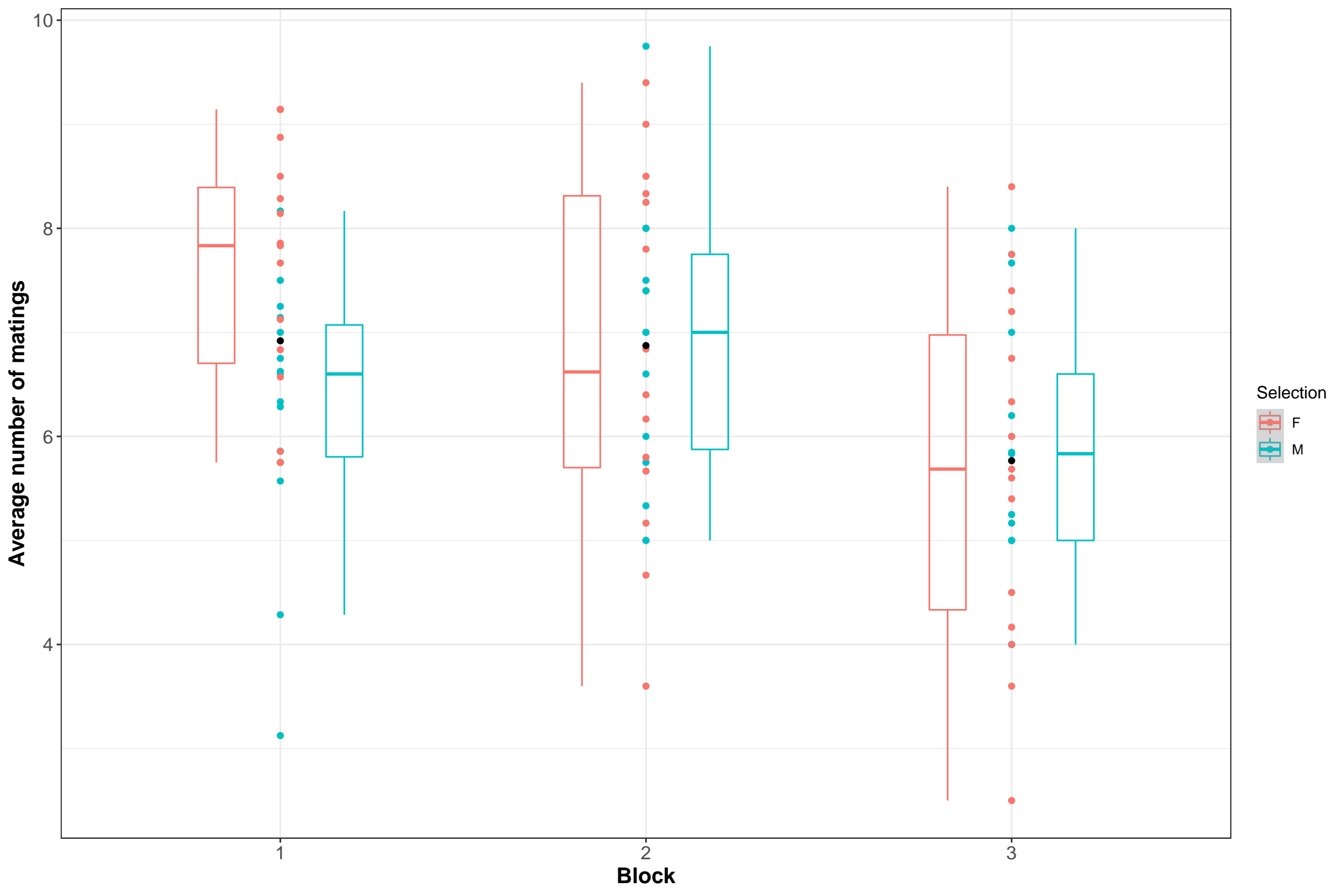

Supplement: Supplementary file 2 — Additional file 2: Fig. S2. Difference in the number of matings acquired by the males between the two selection regimes. [file 12862_2019_1574_MOESM2_ESM.pdf]
